# Pharmacokinetic and Pharmacodynamic Modeling of Enrofloxacin and Its Metabolite Ciprofloxacin in Pregnant Goats

**DOI:** 10.3390/vetsci12060588

**Published:** 2025-06-15

**Authors:** Luis Adrian Ambros, Verónica Kreil, José Julio de Lucas Burneo, Mariano Guillermo Tinti, Manuel Ignacio San Andrés Larrea, Augusto Matías Lorenzutti

**Affiliations:** 1 Cátedra de Farmacología, Facultad de Ciencias Veterinarias, Universidad de Buenos Aires, Buenos Aires C.P. 1427, Argentina; ambros@fvet.uba.ar (L.A.A.); kreil@fvet.uba.ar (V.K.); 2 Instituto en Investigaciones en Producción Animal (INPA), CONICET-Universidad de Buenos Aires, Buenos Aires C.P. 1427, Argentina; 3 Departamento de Farmacología y Toxicología, Facultad de Veterinaria, Universidad Complutense de Madrid, 28040 Madrid, Spain; delucas@vet.ucm.es; 4 Facultad de Ciencias Agropecuarias, IRNASUS CONICET-Universidad Católica de Córdoba, Córdoba X5016DHK, Argentina; 1206209@ucc.edu.ar

**Keywords:** enrofloxacin, ciprofloxacin, goats, gestation, PK/PD modeling

## Abstract

The pharmacokinetics of enrofloxacin and its metabolite ciprofloxacin, as well as the placental transfer of enrofloxacin and ciprofloxacin, have not been studied. The aims of this study were (1) to evaluate the pharmacokinetics of enrofloxacin and ciprofloxacin by intravenous and intramuscular administration of 7.5 mg/kg in pregnant goats; (2) to determine the placental transfer of enrofloxacin and ciprofloxacin; (3) to conduct a PK/PD analysis to calculate the PK/PD cutoff of different dose regimens; and (4) to evaluate the tentative epidemiological cutoff values for coagulase-negative staphylococci wild-type isolates from goats. Plasmatic concentrations of enrofloxacin and ciprofloxacin in pregnant goats were well described by the parent–metabolite model. Simultaneous modeling of enrofloxacin and ciprofloxacin in each individual allowed for a PK/PD analysis that considered both drugs with antimicrobial activity. Our results show that both enrofloxacin and ciprofloxacin crossed the placenta in goats: fetal/maternal concentration ratio were 0.58 ± 0.05 and 0.03 ± 0.01 for enrofloxacin and ciprofloxacin. MIC values of coagulase-negative staphylococci isolates (*n* = 90) were obtained, and tentative epidemiological cutoffs were calculated at 0.25 and 0.5 mg/L for enrofloxacin and ciprofloxacin. According to PK/PDco values, an intravenous dose regimen of 10 mg/kg/day was considered the most appropriate, but based on the PK/PDco, culture, and AST data, an effective dosing regimen with the lowest possible dose could be selected to minimize the potential risk of fetal exposure to enrofloxacin.

## Simple Summary

Pharmacokinetics studies how drugs move in the body between the blood and different organs and tissues. When a treatment is given to a pregnant animal, it is important to consider that the physiological changes during pregnancy can affect how the drug is absorbed, distributed, and eliminated from the body. It is also essential to evaluate whether the drug can reach the fetus. When initiating antimicrobial therapy, it is important to ensure that effective concentrations of the antimicrobial are achieved in the body in order to guarantee the efficacy. The main objective of this study was to model the pharmacokinetics of enrofloxacin and its active metabolite, ciprofloxacin, using mathematical–statistical models (called nonlinear mixed-effects models) and subsequent pharmacokinetic simulation. The results of this study show that both drugs crossed the placenta and reached the fetus, with higher concentrations of enrofloxacin than ciprofloxacin, and conclude that an enrofloxacin dose regimen of 10 mg/kg/day was the most appropriate for the treatment of infections by susceptible microorganisms in pregnant goats. It is important to emphasize that a correct dosage is vital to maximize the effectiveness of the treatment, minimizing the risk of adverse effects in the animals and the emergence of antimicrobial resistance.

## 1. Introduction

In low-income and resource-limited regions, goats constitute a vital component of subsistence livestock systems, providing meet, milk, and leather.

In these production systems, animals undergo various physiological states, growth, pregnancy, and lactation, throughout their productive life. During pregnancy, dosage regimens may require modification to account for changes in drug pharmacokinetics or potential risk of adverse effects on fetal development. Physiological changes, such as decreased plasma protein concentrations, increased intravascular volume, and elevated total body water and fat content, can modify the distribution of many drugs [1,2,3,4,5]. Additionally, the enhanced synthesis of hepatic cytochrome P450 enzymes, along with increased cardiac output, renal blood flow, and glomerular filtration rate (which may rise by up to 50%), can significantly accelerate the elimination of drugs metabolized or excreted through these pathways [1,2,3,4]. However, such changes in the pharmacokinetic profile of a drug are not consistently reported, and the necessity to adjust the dosage regimen may vary accordingly. Regarding the clinical safety of drug use during pregnancy, it is essential to take into account the extent to which a drug crosses the placental barrier, as certain compounds may exert toxic or adverse effects on fetal development.

Placenta is a selective biological barrier with various transporters systems, such P-glycoprotein (P-gp) or multidrug resistance proteins (MRPs), which regulate the exchange of nutrients, gases, wastes, and endogenous and exogenous molecules, including drugs, between maternal and fetal circulation [6,7].

Enrofloxacin (ENR) is a broad-spectrum fluoroquinolone for veterinary use only, active against Gram-negative and Gram-positive aerobic microorganisms. In the organism, ENR is metabolized in variable proportions, depending on the animal species, to ciprofloxacin (CIP), an antimicrobial also effective against several pathogens [8]. Fluoroquinolones are considered a critically important antimicrobial group by the World Organization for Animal Health [9]. In the European Union, ENR is classified as a B category antimicrobial. It implies that it only can be used when category C and D antimicrobials are clinically ineffective, and its use should be based on the results of antibiotic susceptibility testing (AST) [10]. One of the most significant toxicological concerns associated with the use of these drugs in growing animals is the induction of erosive arthropathy, articular cartilage degeneration, and other forms of tendon injury; despite this, a study in pregnant mares suggested that ENR administration to late-term pregnancy did not result in toxicity for the fetuses, although, this might be taken with precaution because of cross-species extrapolation [11].

In vivo, the ratio of fetal blood to maternal blood concentrations in humans has been studied for [12], clindamycin [13], moxifloxacin, levofloxacin, cefepime, and cefoperazone [14]. In vitro studies with perfused human placenta reported a low passage of several fluoroquinoles [15]. Quantification of drug transfer across the placenta could inform pharmacological decisions aimed at minimizing or targeting fetal drug delivery.

The pharmacokinetics of ENR or ENR and its metabolite CIP have been reported following intravenous and intramuscular administration in goats [16,17,18,19,20,21,22]. To the authors’ knowledge, the pharmacokinetics of ENR and its metabolite CIP after intravenous (IV) and intramuscular (IM) administration in pregnant goats, as well as the placental transfer of enrofloxacin and ciprofloxacin in this species, have not been studied.

Nonlinear mixed-effects (NLME) models are a powerful tool used to conduct PK/PD analysis of antimicrobials, since they allow a robust estimation of model parameters and inter-individual variability (IIV). Additionally, this approach can evaluate the effect of covariates on the variability of the model parameters [23,24]. In this way, NLME models are capable of simultaneous modeling of a parent drug and its metabolites, allowing for us to conduct further PK/PD simulations including both parent and metabolite drugs. Since both ENR and CIP have antimicrobial activity, CIP concentrations should be taken into account for PK/PD analysis.

The aims of this study were (1) to evaluate the pharmacokinetic profile of ENRs and their metabolite CIP after intravenous and intramuscular administration in pregnant goats by a parent–metabolite NLME model; (2) to assess the transplacental passage of ENR and CIP following intravenous administration; (3) to perform a PK/PD analysis from the final pharmacokinetic model in order to determine the PK/PD cutoff of different dose regimens; and (4) to evaluate the tentative epidemiological cutoff values for coagulase-negative staphylococci wild-type isolates from goats.

## 2. Materials and Methods

### 2.1. Animals

Five pregnant goats in their first or second pregnancy were included in this study; mean body weight of 46.50 ± 2.29 kg and age range between 2 and 3 years. Each animal was carrying twin fetuses. The pregnancy was diagnosed by ultrasound. All animals were clinically healthy based on their medical records, thorough physical assessment (including evaluation of body temperature, heart, and respiratory frequencies), as well as hematological and biochemical analyses. During a three-week acclimatization period and throughout the experimental protocol, the animals were maintained in a shelter situated within the grounds of the Faculty of Veterinary Sciences at the University of Buenos Aires (FCV-UBA), Buenos Aires, Argentina, with access to natural pasture, fresh forage, and water ad libitum. The animals did not receive antibiotic treatment at least one month before the experiment. All animal procedures were approved by the Institutional Animal Care and Use Committee of the Faculty of Veterinary Science (Protocol N° 2010/22).

### 2.2. Experimental Design

A parenteral formulation of ENR 5% (Floxagen^®^, Laboratorio Vetanco, Villa Martelli, Vicente Lopez, Provincia de Buenos Aires, Argentina) was used. A single dose of 7.5 mg/kg was administered IV via the right jugular vein and IM into the semitendinosus muscle, in a two-period randomized crossover design (3 IV and 2 IM on days 89–91 of gestation and 2 IV and 3 IM on days 99–101). The washout period between treatments was ten days.

Heparinized blood samples were collected from the left jugular vein at 0, 0.08, 0.17, 0.25, 0.33, 0.5, 0.75, 1, 1.5, 2, 2.5, 3, 4, 6, 8, 10, 12, and 24 h after the IV administration and at 0, 0.17, 0.33, 0.5, 0.75, 1, 1.5, 2, 2.5, 3, 4, 6, 8, 10, 12, and 24 h after the IM drug administration. The sampling times were selected in order to characterize the absorption, distribution, and elimination phases of the parent drug, as well as the biotransformation to its metabolite ciprofloxacin.

Blood samples were centrifuged at 2500 g for 10 min. The plasma obtained was stored at −20 °C until analysis. All samples were assayed no later than 3 months after collection.

### 2.3. Analytical Method

Plasmatic ENR and CIP concentrations were determined by HPLC according to the technique described by de Lucas et al. (2004) [25]. ENR was provided by Laboratorio Vetanco (Villa Martelli, Vicente Lopez, Provincia de Buenos Aires, Argentina) and Ofloxacin (used as internal standard) by Sigma Chemical.(3050 Spruce St Louis MO).

A total of 4.5 mL of trichloromethane was added to 0.3 mL of sample containing 75 µL of a 12.5 µg/mL ofloxacin solution. The contents of the tube were mixed and then centrifuged at 1000 rpm for 7 min. The supernatant was dried and reconstituted with 50 µL of mobile phase and was injected onto an HPLC system.

HPLC determinations were prepared under the following chromatographic conditions: Kromasil 100 C18 5 μm 150 × 4.6 mm column and a Kromasil C18 5 μm 30 × 4.6 mm guard column. The mobile phase consisted of buffer pH 2.7, methanol, acetonitrile, acetic acid, and trimethylamine (74:20:4:1:1 *v*/*v*/*v*/*v*/*v*). The buffer pH 2.7 was a 0.4% aqueous solution of tetrabutylammonium hydrogen sulphate (*p*/*v*) and diammonium hydrogen phosphate (*p*/*v*). The UV detection wavelength was 279 nm, and the flow rate was 1.0 mL/min.

ENR and CIP standard curves were linear between 0.025 and 16.7 µg/mL, and the low limit of quantification (LLOQ) was 0.025 µg/mL for both antimicrobials. Intra-day and inter-day LLOQ coefficients of variation were 1.6% and 2.3%, respectively.

### 2.4. Enrofloxacin and Ciprofloxacin Transplacental Passage

At 145–147 days of gestation, the same five goats (mean body weight: 56.13 ± 6.99 kg) underwent cesarean delivery in the surgical suite of FCV-UBA. Animals received premedication with xylazine (0.01 mg/kg; Xilacina 100^®^, Laboratorio Richmond División Veterinaria, Grand Bourg, Malvinas Argentinas, Provincia de Buenos Aires, Argentina) and a total dose of 50 mg diazepam (Diazepam Lamar^®^, Laboratorios Lamar, Virreyes, San Fernando, Provincia de Buenos Aires, Argentina). A bolus of propofol (5 mg/kg; Propofol^®^, Laboratorio Richmond División Veterinaria, Grand Bourg, Malvinas Argentinas, Provincia de Buenos Aires, Argentina) was administered as the hypnotic agent. For intraoperative analgesia, a spinal anesthetic block was performed using 8 mL of 2% lidocaine (Lidocaine^®^, Laboratorio Richmond División Veterinaria, Grand Bourg, Malvinas Argentinas, Provincia de Buenos Aires, Argentina), applied five minutes before the surgical incision. A dose of 7.5 mg/kg of ENR was administered intravenously, three minutes prior to skin incision. Fetal blood samples (*n* = 10), obtained from the umbilical vein of each fetus (twin births), and maternal blood samples (*n* = 5), collected from the jugular vein, were obtained during surgery within 15 min following drug administration and plasma ENR and CIP concentrations were measured as described in the experimental design of the pharmacokinetic experience. The fetal and maternal plasmatic concentration ratio of ENR and CIP (Concentration_fetus_/Concentration_mother_) was calculated.

### 2.5. MIC and Tentative Epidemiological Cutoff Determination

Ninety coagulase-negative staphylococci (CNS) were isolated from different infectious processes of goats. MIC was established by a serial twofold broth microdilution method according to the guidelines of the Clinical and Laboratory Standards Institute [26] using *Staphylococcus aureus* ATCC 29213 as quality assurance. Since no epidemiological cutoffs (ECOFFs) of ENR nor CIP have been stablished for CNS in goats, tentative ECOFFs (TECOFF) were determined with ECOFFinder version 2.0 [27].

### 2.6. Pharmacokinetic Modeling

Pharmacokinetic analysis was conducted with a nonlinear mixed-effects model (NLME) with Monolix 2024R1^®^ suite software (Simulations Plus/Lixoft, Ltd., Lancaster, CA, USA). Concentration vs. time profiles of enrofloxacin and ciprofloxacin administered by IV and IM route in goats were simultaneously modeled with a parent–metabolite model with intravascular and extravascular routes of administration. Different models of metabolite production were tested: a model with a linear (first order) process and a Michaelis–Menten kinetics model. The model that best fit the data was a bi-compartmental model for ENR with Michaelis–Menten kinetics and a mono-compartmental model for CIP. A multiplicative error model for both ENR and CIP was selected. A free unbound fraction of plasma concentration of ENR was integrated in the ordinary differential equations (ODEs) of the model, because it is the fraction that can diffuse to the peripheral compartment, be taken by hepatocytes to produce CIP, or be excreted from the central compartment. Since protein binding of ENR in goats was not reported and the unbound fraction reported in different species is variable, the unbound fraction of ENR in each individual was estimated as a model parameter. The final model was determined according to the reduced variability, goodness-of-fit plots, the likelihood ratio tests, such as −2⋅log-likelihood (−2LL), Akaike information criterion (AIC), and the Bayesian information criterion (BIC) [24,28].

**Figure 1:**
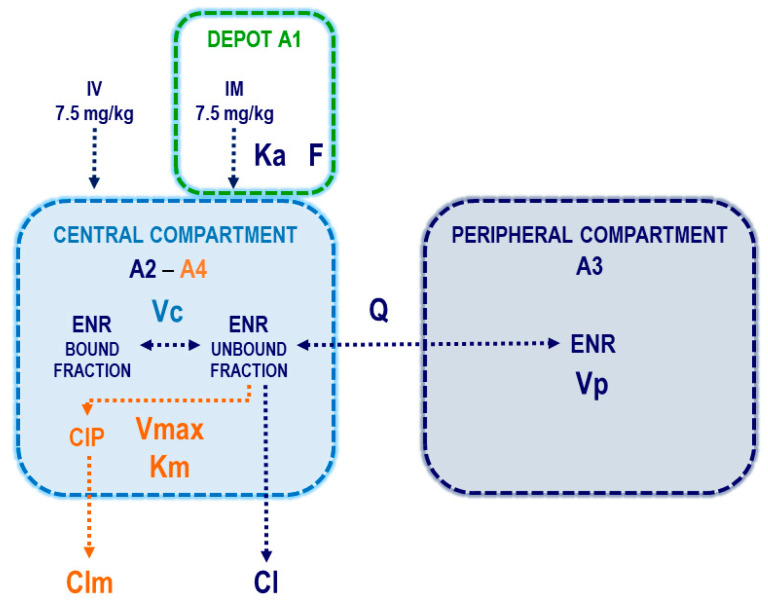
Schematic diagram of the model selected to describe enrofloxacin and its metabolite ciprofloxacin plasmatic concentrations after intravenous and intramuscular administration of 7.5 mg/kg in pregnant goats. Pharmacokinetic parameters of enrofloxacin are shown in blue, ciprofloxacin in orange, depot compartment (administration site of IM dose of ENR) in green, volume of the central compartment (used for both enrofloxacin and ciprofloxacin) in light blue, and peripheral compartment (for ENR alone) in blue. A1, A2, and A3 correspond to ENR amounts and A4 correspond to CIP amounts.

The PK parameters estimated by the model were Ka (absorption constant), F (bioavailability of the IM route), Vc (volume of distribution of the central compartment), Cl (clearance from the central compartment for ENR), Q (inter-compartmental clearance for ENR) and Vp (volume of distribution of the peripheral compartment for ENR), Clm (clearance from the central compartment for CIP), Vmax (maximum rate of CIP production) and Km (Michaelis constant of CIP). A schematic diagram of the model is shown in Figure 1. The ordinary differential equations system used to model ENR and CIP concentrations is shown in Equations (1)–(4):

(1) $$\frac{dA1}{dt}=-Ka\cdot A1$$

(2) $$\frac{dA2}{dt}=Ka\cdot A1-\frac{Cl}{Vc}\cdot A2\cdot fu-\frac{Q}{Vc}\cdot A2\cdot fu+\frac{Q}{Vp}\cdot A3$$

(3) $$\frac{dA3}{dt}=\frac{Q}{Vc}\cdot A2\cdot fu-\frac{Q}{Vp}\cdot A3$$

(4) $$\frac{dA4}{dt}=\frac{\left(Vmax\cdot A2\cdot fu\right)}{\left(Km+A2\cdot fu\right)}-\frac{Clm}{Vc}\cdot A4$$

where A1, A2, and A3 correspond to ENR amounts and A4 correspond to CIP amounts. A1 is the amount of ENR in the administration site, A2 is the amount of ENR in the central compartment, A3 is the amount of ENR in the peripheral compartment, and A4 is the amount of CIP in the central compartment. Plasma concentrations were calculated as A2/Vc and A4/Vc for ENR and CIP, respectively. Secondary pharmacokinetic parameters as the area under the concentration time curve (AUC) for ENR, CIP and ENR + CIP, steady-state volume of distribution (Vss), and terminal half-life (t_1/2_) for ENR were included.

The residual unexplained variability of the model (defined as the difference between predicted and observed concentrations) was described by a proportional error model as follows (Equation (5)):

(5) $$CONCobs=CONCpred+\left(b*CONCpred\right)*\varepsilon$$

where CONCobs is the observed concentration, CONCpred is the predicted concentration, and b is the multiplicative component for the residual error, respectively [24].

In order to determine the effect of different covariates on pharmacokinetics of ENR and CIP, individual weight, age, and number of births were evaluated. For weight, age, and number of births (continuous covariates), the relations between covariates and parameters were evaluated as follows (Equation (6)):

(6) $$\theta i=\theta pop*{\left(\frac{COV\theta i}{COVmean}\right)}^{\beta }*e^{\eta \theta \omega }$$

where θi is the parameter of the model estimated for the ith individual; θpop is the population estimate; COVθi is the covariate value for the ith individual; COVmean is the mean value of the covariate for the studied population; β is the regression coefficient; and ηθω is the IIV of the population estimate for the ith individual. Covariates were included if they showed statistical significance (*p* < 0.05) and reduced the IIV, −2xLL, AIC, and BIC values, and were retained in the final model if they resulted in a ≥10 reduction in BIC [24,29].

The model was evaluated at each step of analysis: goodness-of-fit diagnostics were supported by scatter plots of population/individual predicted versus observed concentrations in a logarithmic and arithmetic scale, population/individual-weighted residuals (PWRES and IWRES) versus predictions/time and visual predictive check (VPC), and prediction distribution plots (PDP) [30]. Standard errors of parameter estimates were calculated based on the full variance–covariance matrix, whereas that the correlation matrix was used to detect over-parameterization of the model [24]. Finally, the robustness of the model was verified using a convergence assessment in Monolix, taking into account 500 replicates, the shrinkage value, and a non-parametric bootstrap analysis with a 90% confidence interval.

### 2.7. Pharmacokinetic Simulations and PK/PD Analysis

The final PK model was exported to Simulx, a simulation software included in the Monolix 2024R1 suite software (Simulations Plus/Lixoft, Ltd., Lancaster, CA, USA). Four multi-dose regimens of ENR administered by IV and IM routes at 7.5 and 10 mg/kg/day for four days were simulated (*n* = 5000 individuals per group). For each simulated individual, ENR, CIP, and ENR + CIP plasma concentration–time profiles were obtained, and steady-state AUCs of ENR, CIP, and ENR + CIP were calculated, obtaining steady-state AUCs distributions of ENR, CIP, and ENR + CIP that were used to determine the probability of target attainment (PTA) by Monte Carlo simulation (*n* = 10,000). PTA was calculated for a MIC range of 0.015–8 mg/L and for different AUC/MIC pharmacodynamic targets (PDT): 24, 48, 72, and 96 h. Finally, PK/PD cutoff (PK/PDco), defined as the highest MIC for which 90% of simulated population achieved a determined pharmacodynamic target of the AUC/MIC index, was obtained from these simulations.

## 3. Results

### 3.1. Pharmacokinetic Modeling

**Figure 2:**
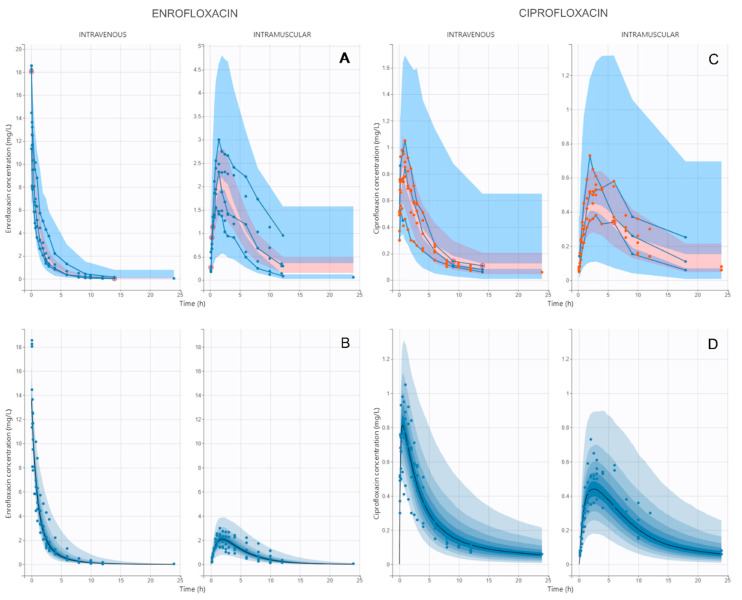
Visual predictive check plot (**A**,**C**) and prediction distribution plots (**B**,**D**) stratified by route of administration for the parent–metabolite model of enrofloxacin and its metabolite ciprofloxacin administered at 7.5 mg/kg by the intravenous and intramuscular route in pregnant goats. The observed and predicted 10th and 90th percentiles of the prediction interval are shown in blue, the 50th percentile (median) is shown in pink, and the observed data are shown as blue or orange dots. For PDPs, median is shown in black and the blue gradient corresponds to 95% prediction interval.

The model that best fitted the pharmacokinetics of ENR and CIP in pregnant goats after IV and IM administration was a bi-compartmental model with first-order absorption for ENR, Michaelis–Menten kinetics for biotransformation from ENR to CIP, a mono-compartmental model for CIP, and a multiplicative error model for both ENR and CIP. VPC and PDP plots stratified by route of administration are presented in Figure 2, showing that most observed data fell into the prediction intervals and were centered around the median. Model parameters are presented in Table 1.

The precision of the estimates was adequate, with CV ˂ 46% in all cases and shrinkage values from −17.2 to 8.05%, indicating that the individual parameters were adequately distributed throughout the population distribution. Goodness-of-fit diagnostic plots and nonparametric bootstrap showed a good fit of the model with the observed data (Figures S1–S6 of Supplementary Materials). None of the evaluated covariates produced a significant effect on model parameters.

### 3.2. Enrofloxacin and Ciprofloxacin Transplacental Passage

ENR and CIP placental transfer was evaluated by calculation of the fetal/maternal plasma concentration ratio after 15 min of ENR administration by intravenous route (7.5 mg/kg). Fetal/maternal ratio was 0.56 ± 0.04 and 0.03 ± 0.01 for ENR and CIP, respectively, indicating a high passage of ENR, but negligible for CIP.

### 3.3. MIC and Tentative Epidemiological Cutoff Determination

Ninety CNSs were isolated from subclinical and clinical mastitis (milk samples), genito-urinary infections (vaginitis), and skin abscesses from goats. MIC_90_ was 0.125 and 0.25 mg/L for ENR and CIP, respectively. A unimodal MIC distribution was observed for ENR and CIP. TECOFF of CNS isolates for ENR and CIP were 0.25 and 0.5 mg/L, respectively (see Figure S7 in Supplementary Materials).

### 3.4. Pharmacokinetic Simulations and PK/PD Analysis

Simulated concentration–time profiles of ENR, CIP, and ENR + CIP after intravenous and intramuscular administration of ENR at a dose of 7.5 and 10 mg/kg (*n* = 5000 each) are presented in Figure S8 of Supplementary Materials. From the simulated dose regimens, steady-state AUCs of ENR and ENR + CIP were obtained (Table 2), and PTAs of each dose regimen were determined by Monte Carlo simulation for the following PDTs: 24, 48, 72, and 96 h and a MIC range from 0.03 to 8 mg/L. Finally, PK/PDco for each dose regimen was calculated (Table 3).

## 4. Discussion

### 4.1. Pharmacokinetic Modeling

In this study, the pharmacokinetic behavior of ENR and its active metabolite CIP after IV and IM administration of ENR (7.5 mg/kg) in pregnant goats was conducted by a parent-metabolite NLME model. Placental transfer of ENR and CIP was also determined. One of the main limitations of the model was the low number of animals included in the study. This is due to animal welfare reasons and the complexity of the sampling design when it comes to assessing placental passage of ENR and CIP. Although an NLME model and Monte Carlo simulation were used for PK and PK/PD analysis, which allow for robust estimation of model parameters and their inter-individual variability, the model used in this study may not fully represent the inter-individual variability observed in the goat population, so future population pharmacokinetic studies with a larger number of animals would be necessary.

The main advantage of the use of a NLME model is to characterize the pharmacokinetics of ENR (parent) and CIP (metabolite) within individuals, in contrast to the traditional two stage analysis [31], where both ENR and CIP concentration–time profiles are analyzed separately and it is difficult to conduct further simulations of dose regimens. Since CIP has antimicrobial activity, it should be considered when antimicrobial efficacy is evaluated in a PK/PD analysis.

After IM administration, ENR presented a complete bioavailability, and volume of distribution (Vc + Vp) exceeded 1 L/kg, according to what was previously reported in goats. Inter-compartmental clearance Q indicated a fast equilibration between blood and peripheral tissues. ENR showed a medium overall body extraction ratio of 0.31, indicating an intermediate total body clearance [16,17,18,19,21,22].

The free unbound fraction of ENR estimated by the model was 0.39, indicating a medium protein binding of ENR in goat plasma of 61%, similar to the reported protein binding of ENR in cows (36–60%) and sheep (69%) [8]. A Michaelis–Menten kinetic model best characterized the biotransformation process of ENR to CIP after IV and IM administration of ENR. The parameters Vmax (maximum metabolic rate) and Km (the concentration corresponding to Vmax/2) determine the concentration of ENR that produces saturation of the hepatic biotransformation system, from which it goes from a first-order process to a zero-order process in the rate of CIP formation. A saturable hepatic metabolism of ENR could be responsible for the observed metabolite ratio (AUC-CIP/AUC-ENR), which was 0.20 and 0.47 for IV and IM routes, similar to that reported by Rao et al. (2001–2002) [18,19]. Initial ENR concentration after IV administration was 16.13 ± 3.16 mg/L, exceeding approximately four times Vmax. Saturation of ENR biotransformation could be responsible of the lower metabolite ratio of the IV route. In this manner, the IM route showed a Cmax of 2.28 ± 0.58 mg/L, indicating that the absorption process of ENR prevents the saturation process observed in the IV route, and therefore, a higher fraction of ENR is metabolized to CIP. In this way, at early times, the rate of CIP formation is clearly lower after IV than IM administration, and although the peak CIP is higher after IV administration, it is not as high as would be expected if the reaction were a first-order process. In this way, a good part of the amount of ENR in the central compartment in early times is excreted by other routes or diffuses to the peripheral compartment, and this makes the metabolite ratio lower compared to IM administration, where saturation of the system is not reached and therefore a first-order biotransformation process of ENR to CIP prevails.

Total body extraction ratio was high for CIP (>0.35), indicating a high total body clearance of the metabolite that was apparently higher than reported for goats [32,33]. Approximately 30% of CIP is excreted by renal excretion in goats [34]. Glomerular filtration rate could increase up to 50% during gestation, and this could be a possible explanation for the higher clearance observed in this study for pregnant goats [1,2].

### 4.2. Enrofloxacin and Ciprofloxacin Trasnplacental Passage

The goal of drug use during pregnancy is to treat the mother, the fetus, or both. As the placenta acts as a third compartment, it is important to have a better awareness of the consequences of fetal exposure arising from a pregnant animal treatment.

Our results show that both ENR and its active metabolite CIP crossed the placenta in goats and reached a detectable concentration in the fetuses. However, fetal concentrations reached by the metabolite were much lower than those of the parent drug (the placental transfer of ENR was 18.67 times higher than observed for CIP). In pregnant mares, both the parent drug and the metabolite cross the placenta and they achieve high concentrations in amniotic and allantoic fluids; in turn, ENR and CIP fetal/maternal ratios were higher than our results [11]. However, differences in the experimental design between studies (mainly treatment duration and sampling time) make the results not comparable. Noergaard et al. (2021) [35] reported that in an ex vivo model, CIP crossed the placenta at a slow, constant rate, indicating moderate fetal exposure. The fetal-to-maternal concentration ratio for CIP reported was highly comparable to the EFX fetal-to-maternal ratio observed in this study, with values of 0.53 and 0.58, respectively. Several studies have shown that fluoroquinolones do not pass freely through organ barriers because they are substrates for multiple transporters of the ATP binding cassette in various tissues [36,37]. The placenta has a protective function on fetuses, therefore, it expresses several transporters capable of limiting the passage of drugs. The passage of the ENR and CIP through the goat placenta reported in the present study requires evaluating the possibility of fetal toxicity.

Despite structural differences between the placenta of goats and that of women, the transfer of drugs occurs mainly through passive diffusion. This process is governed by their lipid solubility, dissociation constant (pKa), molecular size, and affinity for maternal and fetal plasma proteins rather than the number of placental layers traversed [38,39,40]. Nonetheless, the placenta plays a crucial protective role for the fetus, employing various mechanisms to restrict the entry of numerous substances. These include metabolizing enzymes and transporter proteins, such as P-glycoprotein, multidrug resistance-associated proteins, and breast cancer resistance proteins, which collectively influence the transplacental movement of a wide range of pharmacological agents [36,37].

### 4.3. Pharmacokinetic Simulations and PK/PD Analysis

PK/PDco of ENR and ENR + CIP were calculated from steady-state AUCs obtained from dose regimen simulations of ENR administered by IV and IM routes at 7.5 and 10 mg/kg/day. It is generally accepted that the AUC/MIC pharmacodynamic target for fluoroquinolones of 33–55 h and 60–100 h is related with adequate efficacy for Gram-positive and Gram-negative pathogens, respectively [29,41]. These PDTs are similar to that used for PK/PDco calculation in this study: 24–48 h for Gram-positive and 72–96 h for Gram-negative pathogens.

When only ENR AUCs were taken into account, for Gram-positive pathogens (PDT 24–48 h), intramuscular dose regimens of 7.5 and 10 mg/kg showed similar efficacy for MIC values ≤ 0.25 mg/L, but an intravenous administration should be recommended for MIC values ≤ 0.5 mg/L. Different results were observed for Gram-negative pathogens (PDT 72–96 h), where only a 10 mg/kg dose regimen presented a good efficacy for MIC values ≤ 0.125 mg/L by the intramuscular route and ≤ 0.25 mg/L by the intravenous route.

Furthermore, when ENR + CIP AUCs were considered, recommended dose regimens for Gram-positive pathogens (PDT 24–48 h) were the intramuscular administration of 7.5 mg/kg for MIC values ≤ 0.25 mg/L, 7.5 mg/kg by the IV route or 10 mg/kg by the IM route for MIC values ≤ 0. 5 mg/L and 10 mg/kg by the IV route for MIC values ≤ 1 mg/L. For Gram-negative pathogens (PDT 72–96 h), the recommended dose regimens were 7.5 or 10 mg/kg by the IM route for MIC values ≤ 0.125 mg/L and 7.5 or 10 mg/kg by the IV route for MIC values ≤ 0.25 mg/L.

As can be seen in Table 3, both intravenous dosing regimens (7.5 and 10 mg/kg) as well as 10 mg/kg IM showed adequate efficacy against the TECOFFs calculated for CNS isolates when ENR and CIP concentrations were taken into account.

Finally, based on the PK/PDco obtained in this study, the 10 mg/kg/day dose regimen was considered the most appropriate, as it demonstrated efficacy against the highest MIC values when both ENR and CIP concentrations were considered. However, since these dose regimens were proposed for pregnant goats, and considering that currently the use of fluoroquinolones in animals should be based on culture and AST data, the dose regimen that demonstrated efficacy with the lowest possible dose for the isolated pathogen (depending on whether it is Gram-positive or Gram-negative) with a “susceptible” (MIC less than or equal to 0.25 mg/L) or “intermediate” (MIC between 0.5 and 1 mg/L) profile according to CLSI (2013) [42] could be selected in order to achieve a good efficacy criteria while minimizing ENR exposure to the fetus, and thus the risk of toxicity.

## 5. Conclusions

ENR and CIP concentrations in pregnant goats were well described by the parent-metabolite model with Michaelis–Menten kinetics for ENR biotransformation. Simultaneous modeling of ENR and CIP in each individual allowed for a PK/PD analysis that considered both drugs with antimicrobial activity. Based on the PK/PDco obtained in this study and the culture and AST data, an effective dosing regimen with the lowest possible dose could be selected to minimize the potential risk of fetal exposure to ENR. Further studies would be needed to evaluate the dosage regimens proposed in this study with a larger number of animals or with subpopulations with different physiological or productive characteristics (non-pregnant or lactating).

## Figures and Tables

**Table 1:** Pharmacokinetic parameters in plasma for enrofloxacin and ciprofloxacin after intravenous and intramuscular administration of 7.5 mg/kg in pregnant goats.

Fixed Effects	Estimate	IIV (CV %)	Bootstrap Estimate (95% CI)	Schrinkage (%)
Enrofloxacin (parent)				
F	0.80	9.97	0.59–0.92	0.20
Ka (1/h)	0.25	25.81	0.19–0.33	0.20
fu	0.39	11.67	0.30–0.84	4.65
Cl (L/h/kg)	0.81	16.28	0.43–1.52	−4.42
Vc (L/kg)	0.65	-	0.56–0.75	-
Q (L/h/kg)	0.28	-	0.15–0.54	-
Vp (L/kg)	2.83	45.79	1.27–6.3	8.05
Ciprofloxacin (metabolite)				
Clm (L/h/kg)	4.56	25.18	3.45–6.04	−1.21
Vmax (mg/L/h)	4.61	1.65	3.62–5.86	−17.2
Km (mg/L)	0.86	13.06	0.45–1.63	6.34
Residual error model				
Enrofloxacin	0.37	-	0.366–0.367	
Ciprofloxacin	0.18	-	0.177–0.186	
Secondary parameters				
Enrofloxacin (parent)				
	Intravenous		Intramuscular	
	Estimate	CV (%)	Estimate	CV (%)
AUC (mg/L/h)	21.02	37.12	14.10	40.34
Vss (L/kg)	3.65	21.90	4.45	33.83
t_1/2_e (h)	3.15	26.89	3.07	32.60
Ciprofloxacin (metabolite)				
	Intravenous		Intramuscular	
	Estimate	CV (%)	Estimate	CV (%)
AUC (mg/L/h)	3.95	25.28	5.80	25.94
MR	0.20	33.08	0.47	64.43

F: bioavailability of IM route; Ka: absorption constant; Vc: volume of distribution of the central compartment; Cl: clearance from the central compartment for ENR; Q: inter-compartmental clearance for ENR; Vp: volume of distribution of the peripheral compartment for ENR; Clm: clearance from the central compartment for CIP; Vmax: maximum rate of CIP production; Km: Michaelis constant of CIP; CI: confidence interval; IIV: inter-individual variability expressed as the coefficient of variation of the random effects; a and b are the components of the error model that describe the residual variability between observed and predicted concentrations; Vss: volume of distribution at steady state; AUC: area under the concentration time curve; t_1/2_e plasma terminal half-life; and MR: metabolite ratio (AUCmetabolite/AUCparent).

**Table 2:** Steady-state AUCs of enrofloxacin and enrofloxacin + ciprofloxacin for the four simulated dose regimens of enrofloxacin administered by intravenous and intramuscular routes in pregnant goats.

	Enrofloxacin			Enrofloxacin + Ciprofloxacin		
	P05	Median	P95	P05	Median	P95
7.5 mg/kg/day IV	14.22	19.11	34.84	17.71	24.74	42.84
7.5 mg/kg/day IM	6.52	13.98	25.91	9.37	19.74	35.95
10 mg/kg/day IV	18.97	25.48	46.45	23.1	32.15	55.79
10 mg/kg/day IM	8.69	18.64	34.54	12.31	25.72	46.38

Steady-state AUCs are expressed as mg/kg/h; P05: 5% percentile; and P95: 95% percentile.

**Table 3:** PK/PDco values for different pharmacodynamic targets of enrofloxacin and enrofloxacin + ciprofloxacin following different intravenous and intramuscular administration of 7.5 and 10 mg/kg/day in pregnant goats. Data expressed as MIC (mg/L) to achieve a certain PDT in the simulated population using a PTA = 90%.

PDT (h)	Dose Regimen			
	7.5 mg/kg/day IV	7.5 mg/kg/day IM	10 mg/kg/day IV	10 mg/kg/day IM
Enrofloxacin				
24	0.5	0.25	0.5	0.25
48	0.25	0.125	0.25	0.125
72	0.125	0.06	0.25	0.125
96	0.125	0.06	0.125	0.06
Enrofloxacin + ciprofloxacin				
24	0.5	0.25	1	0.5
48	0.25	0.125	0.5	0.25
72	0.25	0.125	0.25	0.125
96	0.125	0.06	0.25	0.125

## Data Availability

The data presented in this study are available on request from the corresponding author due to (privacy and legal reasons).

## Supplementary Materials

The following supporting information can be downloaded at: https://www.mdpi.com/article/10.3390/vetsci12060588/s1, Figure S1: Predicted versus observed plot of PK model of ENROFLOXACIN; Figure S2: Predicted versus observed plot of PK model of CIPROFLOXACIN; Figure S3: Population/individual-weighted residuals (PWRES and IWRES) versus predictions/time of ENROFLOXACIN; Figure S4: Population/individual-weighted residuals (PWRES and IWRES) versus predictions/time of CIPROFLOXACIN; Figure S5: Distribution plots of the residuals of ENROFLOXACIN; Figure S6: Distribution plots of the residuals of CIPROFLOXACIN; Figure S7: MIC distributions and TECCOF values for ENROFLOXACIN and CIPROFLOXACIN of coagulase-negative staphylococci isolated from goats; Figure S8: Simulated concentration-time profiles of ENR, CIP and ENR+CIP.
