# Supplementary material for: Pharmacokinetic and Pharmacodynamic Modeling of Enrofloxacin and Its Metabolite Ciprofloxacin in Pregnant Goats
Source: Vet Sci. 2025 Jun 15;12(6):588. doi: 10.3390/vetsci12060588 (PMC12197546; doi:10.3390/vetsci12060588)
Supplement: Supplementary file 1 [file vetsci-12-00588-s001.zip › vetsci-3653157-supplementary.pdf]

Supplementary material

Pharmacokinetic and pharmacodynamic modeling of enrofloxacin and its metabolite ciprofloxacin in pregnant goats

NLME model in MLXTRAN code.

```
DESCRIPTION: PARENT-METABOLITE MODEL WITH MICHAELIS-MENTEN KINETICS OF
PLASMA CONCENTRATIONS OF ENROFLOXACIN AND CIPROFLOXACIN AFTER IV AND IM
ADMINISTRATION OF 7.5 MG/KG IN PREGNANT GOATS

[LONGITUDINAL]

input = {F, ka, fu, Vc, Cl, Q, Vp, Clm, Vmax, Kmax}

PK:

depot(adm=1, target=A2) ; IV administration

depot(adm=2, target=A1, p=F) ; IM administration

EQUATION:

odeType = stiff

t_0=0

A1_0=0 ; IM administration compartment

A2_0=0 ; ENR IV central compartment

A3_0=0 ; ENR Peripheral compartment
```

```
A4_0=0 ; CIP IV central compartment

A5_0=0 ; CIP Peripheral compartment


ddt_A1 = - ka*A1

ddt_A2 = - Cl/Vc*A2*(fu) - Q/Vc*A2*(fu) + Q/Vp*A3 + ka*A1

ddt_A3 = Q/Vc*A2*(fu) - Q/Vp*A3

ddt_A4 = - Clm/Vc*A4 + ((Vmax*(A2*(fu)))/(Kmax*Vc+(A2*(fu))))


CcENR = A2/Vc ; Plasma concentrations at central compartment ENR

CcCIP = A4/Vc ; Plasma concentrations at central compartment CIP

Cccomb = CcENR + CcCIP; Combined plasma concentrations at central compartment of ENR + CIP


AUCenr_0 = 0

AUCcip_0 = 0

ddt_AUCenr = CcENR

ddt_AUCcip = CcCIP

ddt_AUCcomb = Cccomb

Vss = Vc + Vp

HL = 0.693*(Vss/Cl)


OUTPUT:

output = {CcENR, CcCIP, Cccomb}

table = {AUCenr, AUCcip, AUCcomb, Vss, HL}
```

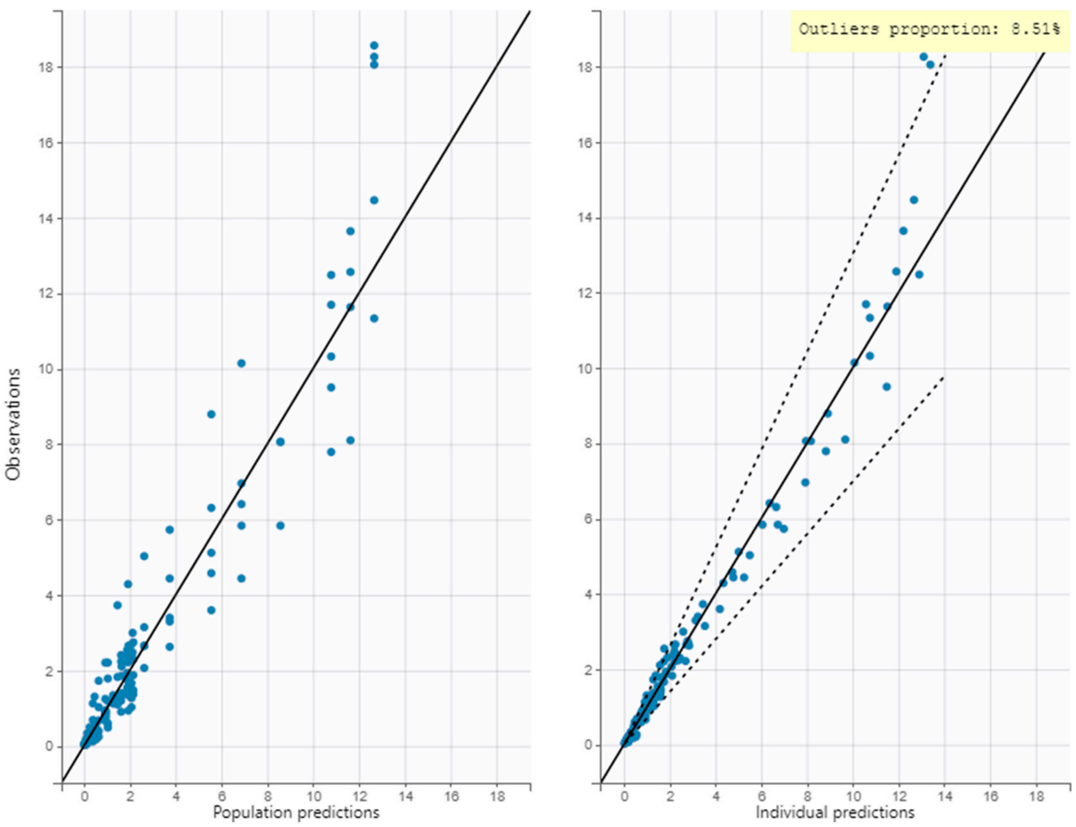

Figure S1: Predicted versus observed plot of PK model of **ENROFLOXACIN**.; plasma concentrations after population and individual predictions.

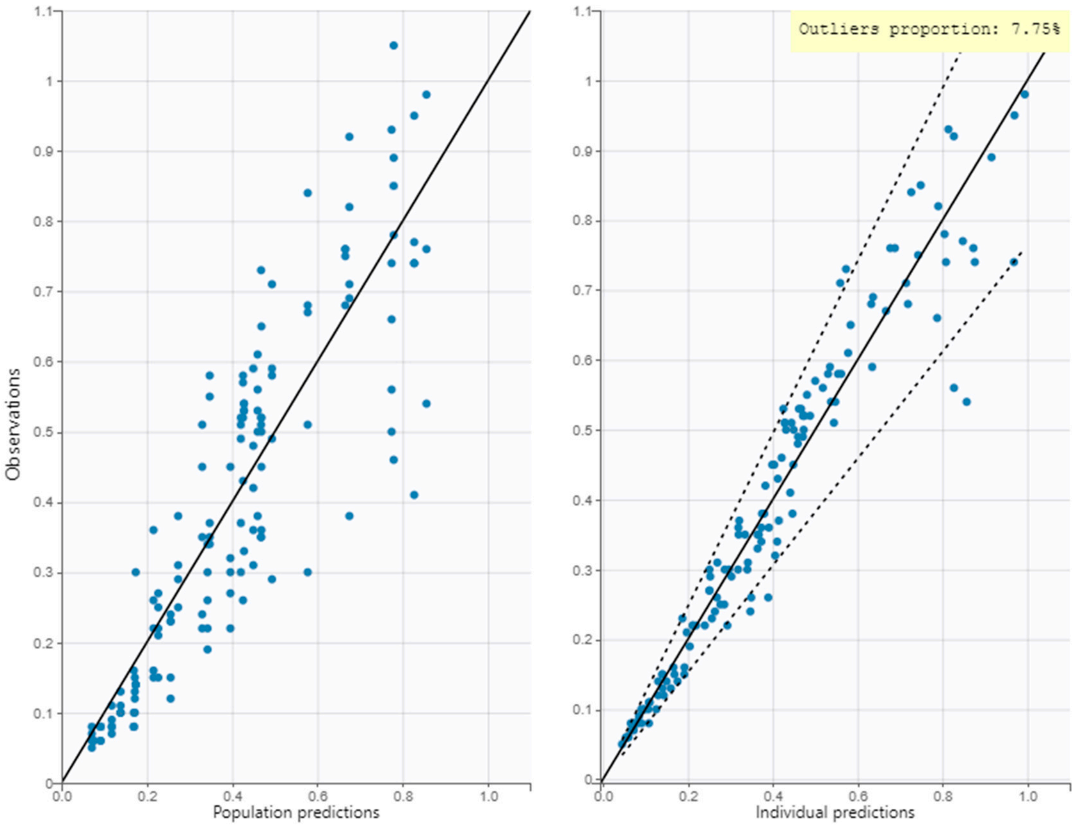

Figure S2: Predicted versus observed plot of PK model of **CIPROFLOXACIN**.; plasma concentrations after population and individual predictions.

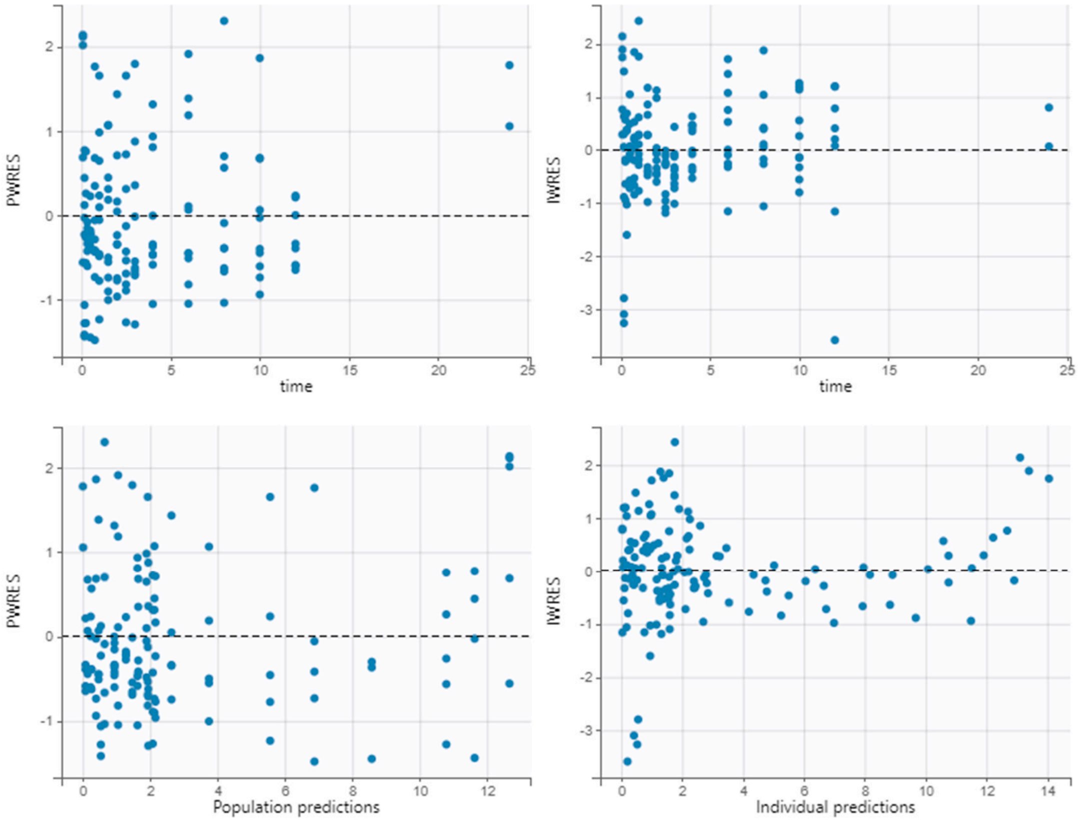

Figure S3: Population/individual-weighted residuals (PWRES and IWRES) versus predictions/time of the **ENROFLOXACIN** PK model.

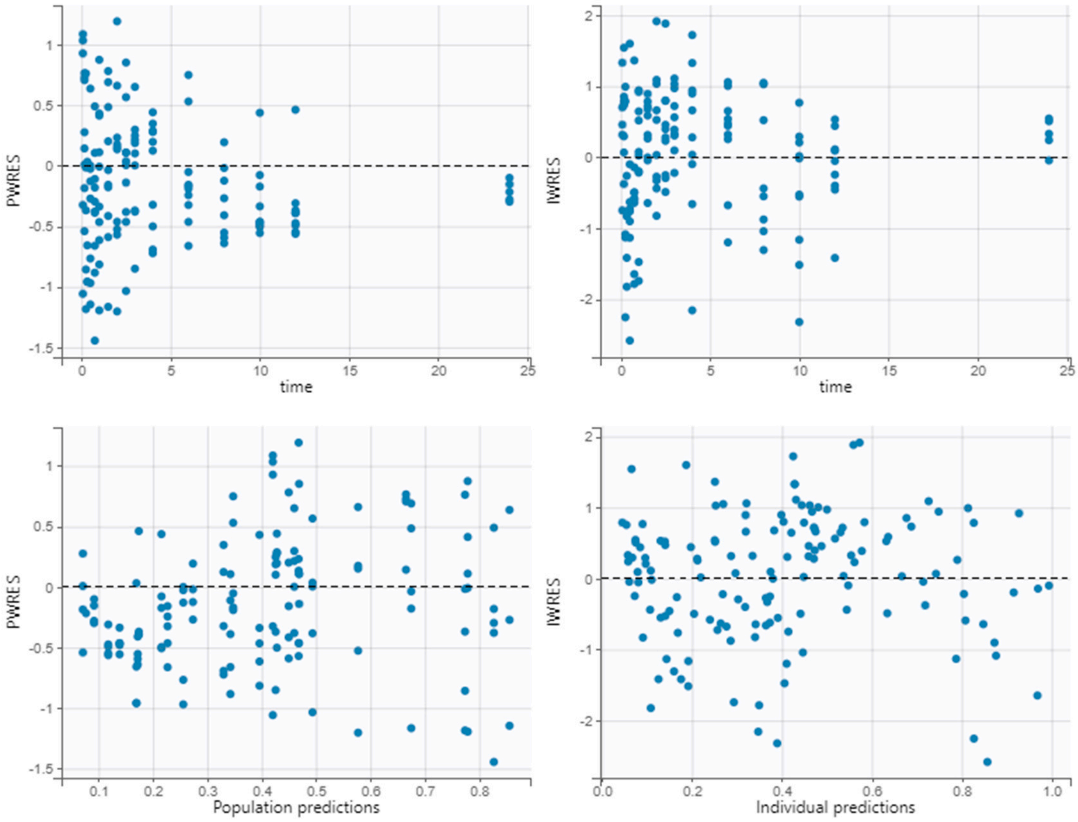

Figure S4: Population/individual-weighted residuals (PWRES and IWRES) versus predictions/time of the **CIPROFLOXACIN** PK model.

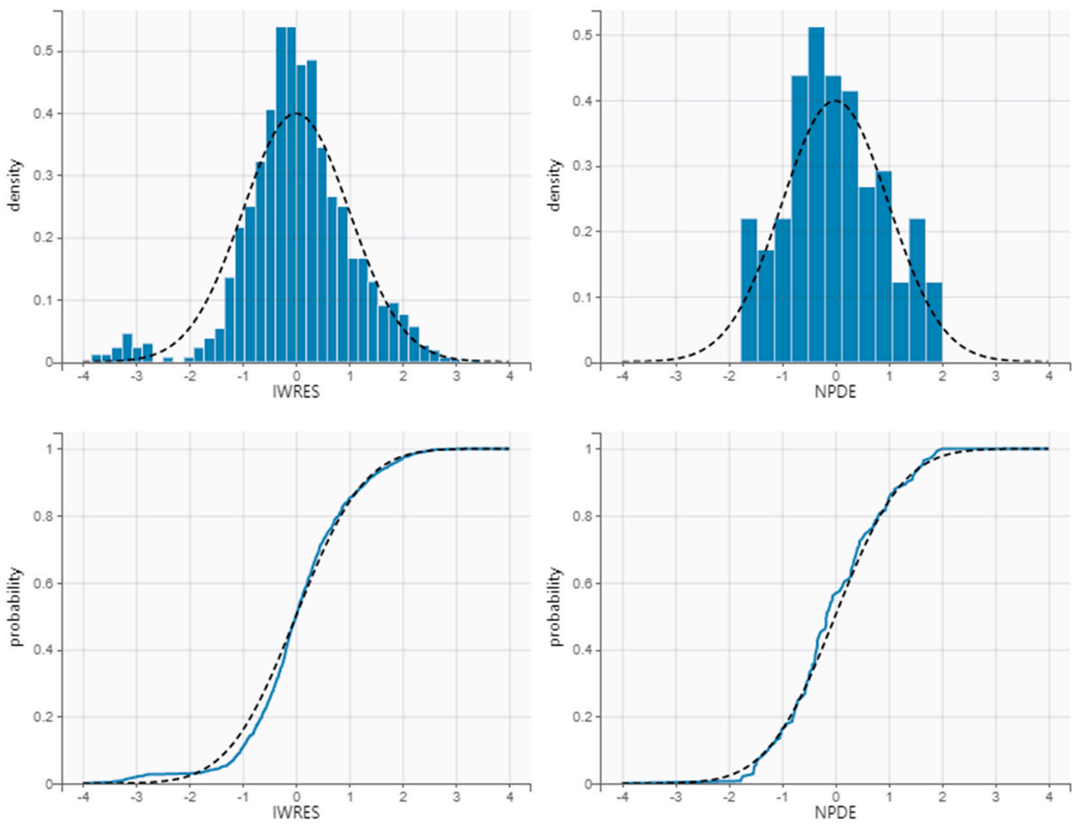

Figure S5: Distribution plots of the residuals for PK model of **ENROFLOXACIN**.

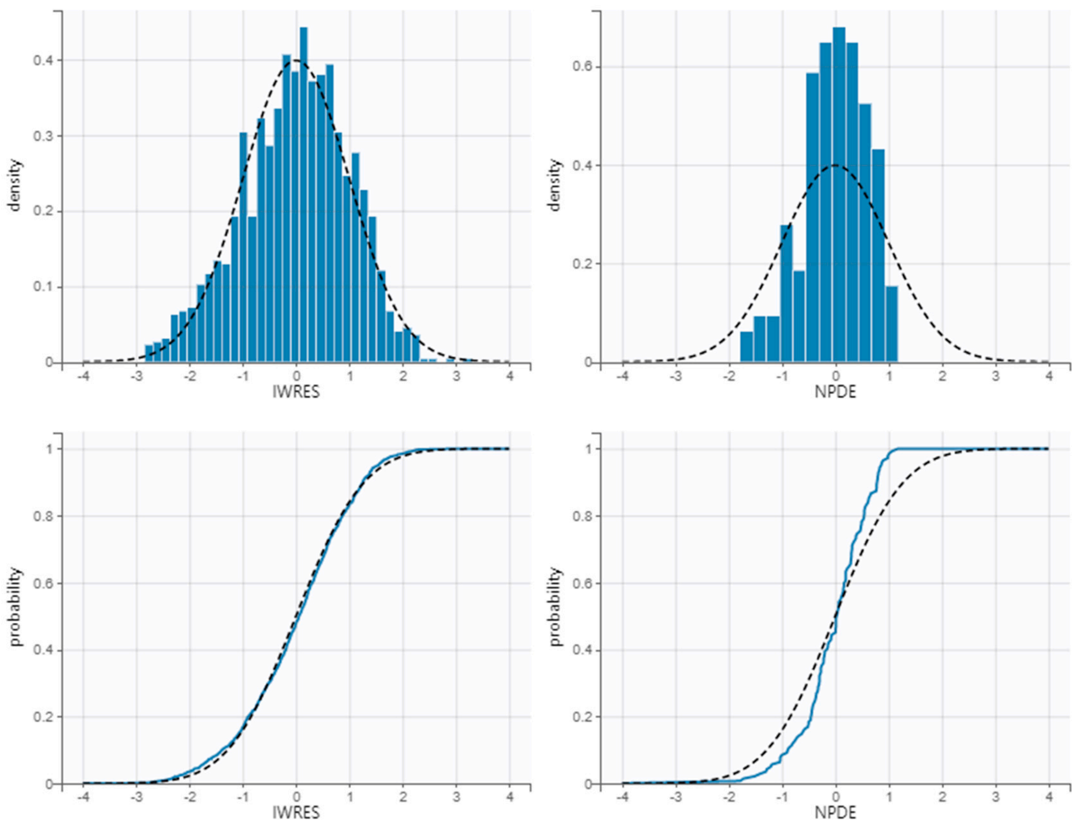

Figure S6: Distribution plots of the residuals for PK model of **CIPROFLOXACIN**.

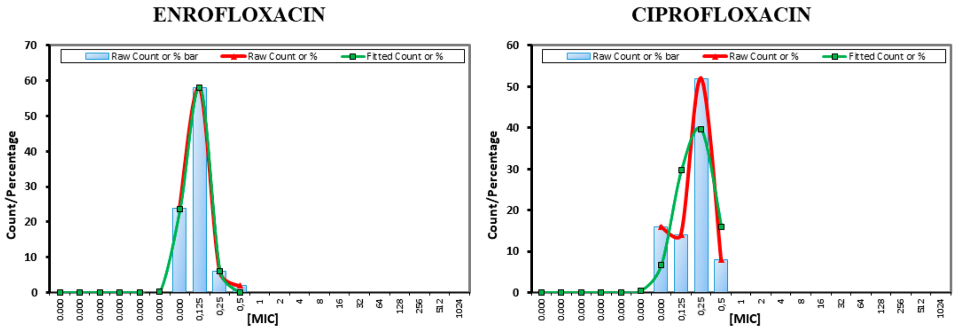

Figure S7: fitted MIC distributions and TECCOF values for ENROFLOXACIN and CIPROFLOXACIN of coagulase-negative staphylococci isolated from goats.

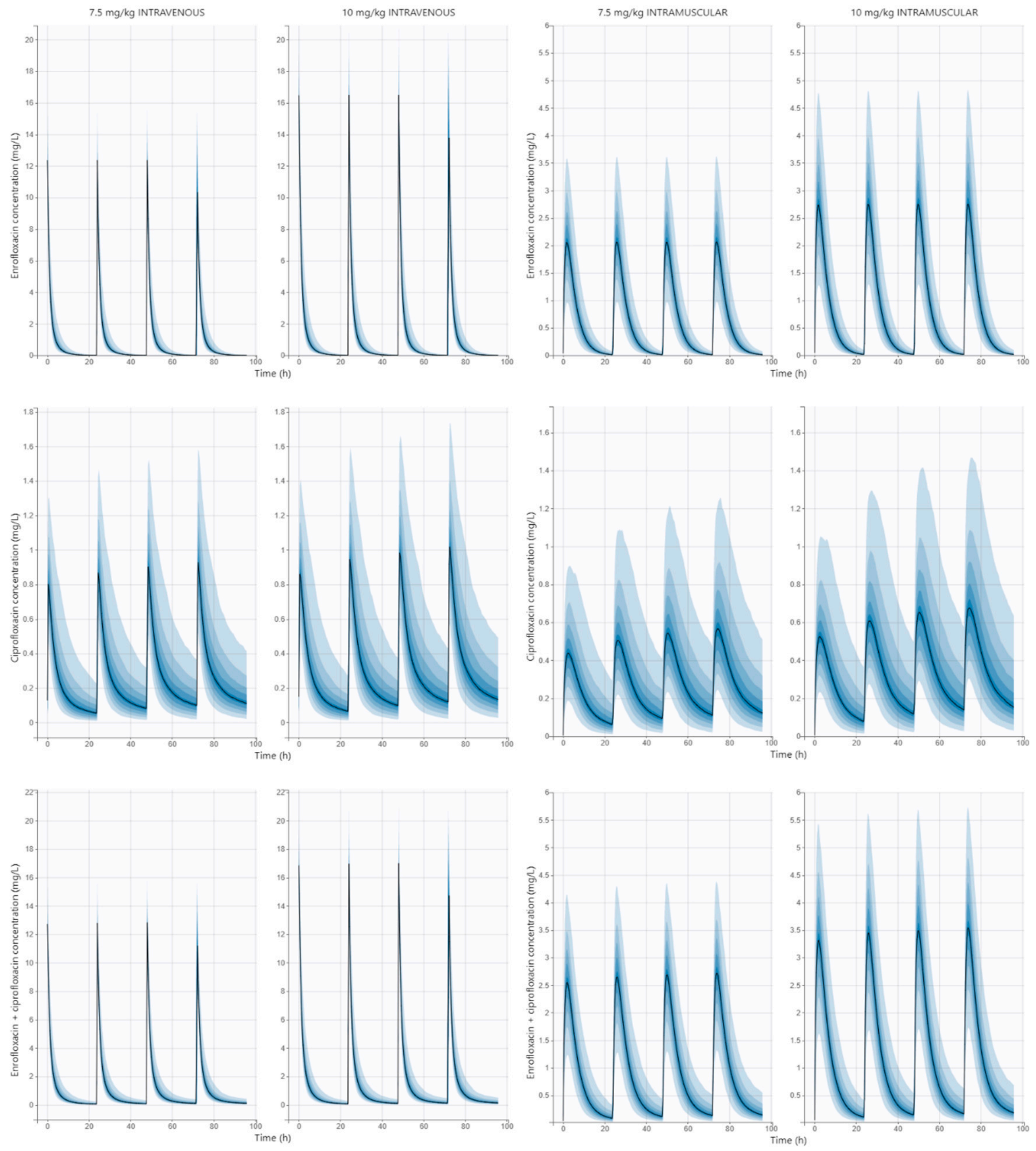

Figure S8: Simulated concentration-time profiles of ENR, CIP and ENR+CIP after intravenous and intramuscular administration of ENR at a dose of 7.5 and 10 mg/kg (n=5000 each).
